# Younger age of onset and uveitis associated with HLA-B27 and delayed diagnosis in Thai patients with axial spondyloarthritis

**DOI:** 10.1038/s41598-021-93001-5

**Published:** 2021-06-29

**Authors:** Naphruet Limsakul, Praveena Chiowchanwisawakit, Parichart Permpikul, Yubolrat Thanaketpaisarn

**Affiliations:** 1 Division of Rheumatology, Department of Medicine, Faculty of Medicine Siriraj Hospital, Mahidol University, Bangkok, Thailand; 2 Vachira Phuket Hospital, Phuket, Thailand; 3 Division of Rheumatology, Department of Medicine, Faculty of Medicine Siriraj Hospital, Mahidol University, 2 Wanglang Road, Bangkoknoi, Bangkok, 10700 Thailand; 4 Department of Transfusion Medicine, Faculty of Medicine Siriraj Hospital, Mahidol University, Bangkok, Thailand

**Keywords:** Clinical genetics, Genetics, Rheumatology

## Abstract

To identify characteristics associated with HLA-B27, and to identify factors associated with delayed diagnosis in Thai patients with axial spondyloarthritis (axSpA). This cross-sectional study included Thai patients were diagnosed with axSpA by a rheumatologist at Siriraj Hospital. Clinical data were collected. Regression analyses were employed to identify factors associated with study outcomes. Of total 177 patients, 127 (72%) were positive HLA-B27. Uveitis [Odds ratio (OR) 4.0], age at onset of the first musculoskeletal symptom of ≤ 28 years [OR 3.5], female [OR 0.4], and psoriasis [OR 0.4] were significantly associated with HLA-B27 in multiple regression analysis. Those with positive HLA-B27 had less spinal flexibility. Elevated C-reactive protein (*p* = 0.012) was associated with shorter delay in diagnosis, while uveitis (*p* < 0.001) and younger age at onset of the first symptom (*p* = 0.002) were associated with longer delay in diagnosis in multiple regression analysis. Younger age at onset of the first musculoskeletal symptom and uveitis were associated with HLA-B27 and delayed diagnosis in axSpA patients. Young people with musculoskeletal symptom and uveitis should be referred to a rheumatologist to rule out or make a timely diagnosis of axSpA.

## Introduction

Spondyloarthritis (SpA) is a group of inflammatory rheumatic diseases. Ankylosing spondylitis (AS) is a type of SpA, and the most common presenting symptom is back pain. Symptom in these patients normally first appears in early adulthood. An average diagnostic delay of 6.7 years^1^ was reported in patients with AS and axial spondyloarthritis (axSpA). Moreover, diagnostic delay was reported to adversely impact the health status of patients with SpA^2,3^. Human leucocyte antigen B27 (HLA-B27) is associated with SpA; however, the strength of association varies among SpA disease spectrum^4^ and ethnicities^5^. The prevalence of HLA-B27 in AS is highest at 90%^6^, followed by reactive arthritis (30–70%), psoriatic arthritis (PsA) (40–50%), and acute anterior uveitis (50%)^6^. HLA-B27 prevalence varies widely around the world from 0 to 53%, and the prevalence of AS is associated with the prevalence of HLA-B27^6^. Positive HLA-B27 was reported to associate with AS range of relative risk of 9.8–43.4^7^. There is conflicting data specific to the characteristics of AS and axSpA patients with HLA-B27^1^. It may be due to polymorphism of HLA-B*27 which associated with different manifestations among ethnicities, various characteristics of study population (disease duration and gender), and different methodologies^1^. However, most previous studies reported younger age at onset of inflammatory back pain (IBP) and shorter delay in diagnosis to be significantly associated with HLA-B27^5,7,8^. Regarding psoriatic arthritis with axial involvement (axial-PsA), knowledge of clinical characteristics, natural history, impact of disease, and response to available treatment is essential; however, it is not clearly understood^9^. AS and axial-PsA have different clinical characteristics has been reported^10^. Patients with axial-PsA are less likely to have IBP and severe disease activity than AS patients^10^; however, it may vary among ethnicities.

In Thailand, the prevalence of HLA-B27 in AS patients was estimated to be 91%^11^. However, no study has investigated the prevalence of and characteristics associated with HLA-B27 in axSpA patients in Thailand. Accordingly, the aims of this study were to estimate the prevalence of HLA-B27, to identify characteristics significantly associated with HLA-B27, and to identify factors significantly associated with delayed diagnosis in Thai patients with axSpA. Our secondary objectives were to identify clinical features that differ significantly between AS and axial-PsA and to estimate the effect of HLA-B27 and delayed diagnosis on clinical outcomes.

## Materials and methods

### Study design and population

This cross-sectional study included Thai patients aged ≥ 18 years that had back pain with a duration at least 3 months of onset before the age of 45 and were diagnosed as axSpA by a rheumatologist at Siriraj Hospital during 31 May 2012 to 31 October 2019. Siriraj Hospital is a 2300-bed university-based national tertiary referral center that is located in Bangkok, Thailand. All eligible patients were invited to join the study, and any refusing musculoskeletal examination, questionnaire completion, pelvis radiography, and/or HLA-B27 testing were excluded. The protocol for this study was approved by the Siriraj Institutional Review Board of the Faculty of Medicine Siriraj Hospital, Mahidol University (COA no Si 247/2012), and written informed consent was obtained from all participants. This study complied with the principles set forth in the 1964 Declaration of Helsinki and all of its subsequent amendments.

### Data collection

Patients were interviewed face-to-face and their medical records were reviewed for baseline characteristics that included age, gender, education, ethnicity, characteristics of back pain and other SpA features according to the Assessment of Spondyloarthritis International Society (ASAS) criteria^12^, date at onset of SpA features, comorbid diseases, and medications. All patients underwent musculoskeletal examination, and each was asked to provide patient report outcome (PRO) data via one or more of the following assessment tools: Bath Ankylosing Spondylitis Disease Activity Index (BASDAI)^13^, Bath Ankylosing Spondylitis Functional Index (BASFI)^13^, the EuroQoL-5 dimensions, 5 levels (EQ5D)^14^. The Thai EQ5D Index^15^ was calculated and scores of 0 and 1.00 are defined as dead and perfect health, respectively. All participants either completed the questionnaire(s) themselves or they were assisted by a trained research assistant.

Physical examination of peripheral joints, including 66 swollen, 68 tender, and 66 damaged joint counts, dactylitis, and enthesitis, was assessed by one rheumatologist (PC). Enthesitis was assessed according to the Spondyloarthritis Research Consortium of Canada Enthesitis Index^16^ and the Maastricht Ankylosing Spondylitis Enthesitis Score^17^. Spinal mobility, as assessed by cervical rotation, tragus to wall distance, occiput to wall distance (OTW), chest expansion, Schober test, and lateral spinal flexion, was assessed following ASAS suggestion^18^ by one rheumatologist (PC), a well-trained nurse (PT), or a fellow in training (NL). Intraclass correlation coefficient of mobility measurement between PC and PT, and PC and NL was excellent at 0.95 [95% confidence interval (CI) 0.94–0.96] and 0.99 [95% CI 0.98–0.99], respectively. Researchers performing physical examinations were blinded to the patient’s questionnaire responses.

HLA- B27 was typed by microlymphocytotoxicity test^19,20^. Serum C-reactive protein (CRP) was measured by particle-enhanced immunoturbidimetric assay (cobas c502 module, cobas 8000 modular analyzer series, Roche Diagnostics International Ltd., Rotkreuz, Switzerland) (normal range 0–5 mg/L).

### Assessment of spondyloarthritis features and classification of spondyloarthritis

SpA features were assessed according to the definitions of the ASAS criteria^12^. The presence or history of the following SpA features was determined: IBP^21^, peripheral arthritis, family history of SpA (family history in first or second-degree relatives of AS, psoriasis, uveitis, reactive arthritis, or inflammatory bowel disease [IBD]), psoriasis, IBD, dactylitis, enthesitis, anterior uveitis, good response to non-steroidal anti-inflammatory drugs (NSAIDs), positive HLA-B27 testing, elevated CRP, and radiographic sacroiliitis (bilateral grade 2–4 or unilateral grade 3–4 according to the modified New York criteria)^22^. No patient underwent magnetic resonance imaging (MRI) of the pelvis in this study. AxSpA was divided by absence or presence of evidence of radiographic sacroiliitis as mentioned above^22^ into non-radiographic axSpA (nr-axSpA) and radiographic axSpA (r-axSpA), respectively. AS was diagnosed by a rheumatologist following the modified New York criteria^22^ and PsA was diagnosed by a rheumatologist and classified following the classification criteria for psoriatic arthritis (CASPAR)^23^. Undifferentiated SpA (uSpA) was diagnosed by a rheumatologist if an axSpA patient could not fulfill the modified New York criteria^22^ and CASPAR^23^.

### Sample size calculation and statistical analysis

Using an established prevalence of 70%^8,24^ and a 7% error, a sample of 165 patients was required. To compensate for missing data, the sample size was increased to 180 patients. Comparison of continuous variables was performed using Student’s *t*-test or Mann–Whitney U-test depending on the pattern of data distribution. Pearson’s χ^2^ test or Fisher’s exact test was used to compare categorical data. To identify clinical features associated with HLA-B27, and differences in clinical features between AS and PsA, and to estimate the effect of HLA-B27 on clinical outcomes, binary logistic regression was performed. Variables with a *p*-value less than 0.1 and other factors of interest were entered into multiple regression analysis. A *p*-value of less than 0.05 was considered statistically significant for all other tests. The adequacy of the logistic regression models was examined using Hosmer–Lemeshow test. To determine the factors associated with diagnostic delay in years, and to estimate effect of diagnostic delay on clinical outcomes using multiple linear regression, diagnostic delay was transformed using log 10 due to positive skewness. Multicollinearity between independent variables was assessed by variance inflation factor (VIF) for multiple linear regression. All statistical analyses were performed using SPSS Statistics version 20.0 (SPSS, Inc., Chicago, IL, USA).

## Results

Two hundred and forty-eight patients were selected. Sixty-eight patients were excluded from the study due to performing only pelvic radiography (53 patients) and only HLA-B27 (15 patients). Most characteristics of included and excluded participants were comparable, except the mean number of SpA features [4.5 vs 4.1, *p* = 0.030] and proportion of having enthesitis [119 (67.2%) vs 35 (51.5%, *p* = 0.022)]. One hundred and seventy-seven patients were recruited. Of those, 67 (37.9%) were female, the mean plus/minus standard deviation age was 39.5 ± 10.4 years; 168 (94.9%) patients were r-axSpA, and 9 (5.1%) patients were nr-axSpA. The prevalence of positive HLA-B27 was 71.8% (Table 1).

**Table 1:** Characteristics of axial spondyloarthritis participants compared between included participants with positive and negative HLA-B27 status.

Characteristics	Included participants			
	Total (n = 177)	Positive HLA-B27 (n = 127)	Negative HLA-B27 (n = 50)	*p-*value^#^
Age (years), mean ± SD	39.5 ± 10.4	39.9 ± 10.5	38.4 ± 9.9	0.404^a^
Female gender, n (%)	67(37.9%)	41 (32.3%)	26 (52.0%)	0.015^c^
First symptom age (years), mean ± SD	28.5 ± 9.6	27.1 ± 9.6	31.9 ± 8.6	0.002^a^
IBP onset (years), mean ± SD	29.5 ± 8.9	28.14 ± 8.6	32.8 ± 8.8	0.002^a^
First axial symptoms, n (%)	122 (68.9%)	94 (74.6%)	28 (56.0%)	0.016^c^
First arthritis-enthesitis, n (%)	72 (40.7%)	47 (37.3%)	25 (50.0%)	0.122^c^
Age at diagnosis (years), mean ± SD	36.1 ± 10.5	36.0 ± 11.0	36.6 ± 9.2	0.722^a^
Delayed diagnosis (years), median (IQR)	5.0 (1.7–11.1)	6.0 (2.0–12.2)	2.5 (0.8–6.3)	0.001^b^
Disease duration (years), median (IQR)	9.6 (3.6–15.7)	10.9 (5.0–26.1)	4.7 (2.1–15.1)	< 0.001^b^
No. of SpA features, mean ± SD	4.5 ± 1.5	4.5 ± 1.5	4.6 ± 1.6	0.837^a^
Inflammatory back pain, n (%)	151 (85.3%)	110 (87.3%)	41 (82.0%)	0.364^c^
Arthritis, n (%)	142 (80.2%)	91 (71.7%)	42 (84.0%)	0.087^c^
Enthesitis, n (%)	119 (67.2%)	84 (66.1%)	35 (70.0%)	0.622^c^
Uveitis, n (%)	43 (24.3%)	39 (30.7%)	4 (8.0%)	0.002^c^
Dactylitis, n (%)	43 (24.3%)	31 (24.4%)	12 (24.0%)	0.954^c^
Psoriasis, n (%)	48 (27.1%)	22 (17.3%)	26 (52.0%)	< 0.001^c^
Inflammatory bowel disease, n (%)	2 (1.1%)	1 (0.8%)	1 (2.0%)	0.486^d^
Good response to NSAIDs, n (%)	91 (51.4%)	66 (52.8%)	25 (50.0%)	0.738^c^
Family history, n (%)	54 (30.5%)	42 (33.1%)	12 (24.0%)	0.238^c^
Elevated C-reactive protein, n (%)	104 (59.4%)	78 (62.4%)	26 (52.0%)	0.206^c^
Radiographic axSpA, n (%)	168 (94.9%)	118 (92.9%)	50 (100%)	0.063^d^
OTW, median (IQR)	0 (0–4.0)	0 (0–6.3)	0 (0–0)	< 0.001^c^
Chest expansion, mean ± SD	3.4 (1.4)	3.2 ± 1.4	3.8 ± 1.3	0.022^a^
Schober test, mean ± SD	3.9 (1.7)	3.7 ± 1.8	4.6 ± 1.3	< 0.001^a^
BASFI, median (IQR)	2.3 90.7–4.9)	2.4 (0.9–5.0)	1.8 (0.4–4.2)	0.069^b^
BASDAI, median (IQR)	3.4 (2.1–5.5)	3.8 (2.1–5.6)	3.9 (1.9–5.4)	0.912^b^
EQ5D index, mean ± SD	0.8 ± 0.2	0.74 ± 0.21	0.77 ± 0.20	0.445^a^

*BASDAI* Bath Ankylosing Spondyloarthritis Disease Activity Index, *BASFI* Bath Ankylosing Spondylitis Functional Index, *EQ-5D index* EuroQoL 5‐dimension 5‐level (EQ‐5D‐5L) index, *Family history* presence in first-degree or second-degree relatives of any of the following: ankylosing spondylitis, psoriasis, uveitis, reactive arthritis, or inflammatory bowel disease, *First axial symptom* first presentation with inflammatory back pain and/or buttock pain, *First arthritis-enthesitis* first presentation with peripheral arthritis or enthesitis, *First symptom age* age at onset of the first musculoskeletal symptoms, *IBP onset* age at onset of inflammatory back pain, *IQR* interquartile range, *Delayed diagnosis* the duration from the first musculoskeletal symptom to diagnosis, *No. of SpA features* the number of spondyloarthritis features, excluding HLA-B27, *NSAIDs* non-steroidal anti-inflammatory drugs, *OTW* occiput to wall distance, *Radiographic axSpA* axial spondyloarthritis with evidence of radiographic sacroiliitis according to the modified New York criteria, *SD* standard deviation.

A *p*-value < 0.05 indicates statistical significance.

^**#**^Compared between positive and negative HLA-B27 included participants.

^a^Unpaired t-test.

^b^Mann–Whitney U-test.

^c^Pearson’s χ^2^ test.

^d^Fisher’s exact test.

### Characteristics of axSpA patients with positive HLA-B27

Patients with positive HLA-B27 were more likely to have the first presentation with axial symptom (IBP or buttock pain), to be younger age at onset of the first musculoskeletal symptom and onset of IBP, to have longer duration of delayed diagnosis, and to have uveitis than those with negative HLA-B27 (Table 1). Conversely, those with positive HLA-B27 were less likely to be female, and to have psoriasis than those with negative HLA-B27; however, the other features were comparable. In multiple logistic regression, age at onset of the first symptom of ≤ 28 years and uveitis were positively associated with HLA-B27 while female, and psoriasis was negatively associated with HLA-B27 among axSpA after adjusted with other variables (Table 2).

**Table 2:** Clinical characteristics associated with HLA-B27 among 177 patients with axial spondyloarthritis.

Characteristics	Univariate logistic regression		Multiple logistic regression	
	OR (95% CI)	*p-*value	OR (95% CI)	*p-*value
Female gender	0.44 (0.23–0.86)	0.016	0.41 (0.19–0.89)	0.025
Age at onset of 28 years	4.53 (2.19–9.36)	< 0.001	3.46 (1.55–7.73)	0.003
First axial symptoms	2.24 (1.13–4.4)	0.021	1.46 (0.61–3.47)	0.393
First arthritis-enthesitis	0.59 (0.30–1.14)	0.115	NA	NA
Inflammatory back pain	1.42 (0.59–3.44)	0.437	NA	NA
Arthritis	0.48 (0.21–1.13)	0.091	0.69 (0.26–1.85)	0.464
Enthesitis	0.84 (0.41–1.70)	0.623	NA	NA
Uveitis	5.10 (1.72–15.14)	0.003	4.02 (1.23–13.20)	0.022
Dactylitis	1.023 (0.48–2.20)	0.954	NA	NA
Psoriasis	0.19 (0.09–0.40)	< 0.001	0.38 (0.16–0.90)	0.028
Inflammatory bowel disease	0.0 (0.02–6.34)	0.507	NA	NA
Good response to NSAIDs	1.08 (0.56–2.08)	0.814	NA	NA
Family history	1.57 (0.74–3.30)	0.240	NA	NA
Elevated C-reactive protein	1.53 (0.79–2.97)	0.207	NA	NA

*Age at onset of 28 years* age at onset of the first musculoskeletal symptoms of ≤ 28 years, *Family history* presence in first-degree or second-degree relatives of any of the following: ankylosing spondylitis, psoriasis, uveitis, reactive arthritis, or inflammatory bowel disease, *First axial symptom* first presentation with inflammatory back pain and/or buttock pain, *First arthritis-enthesitis,* first presentation with peripheral arthritis or enthesitis, *NA* not applicable, *NSAIDs* non-steroidal anti-inflammatory drug.

A *p*-value < 0.05 indicates statistical significance; logistic regression analysis was used to identify significantly associated factors with HLA-B27 among axial spondyloarthritis; the dependent factor was axial spondyloarthritis with positive HLA-B27.

Patients with positive HLA-B27 were more likely to have less spinal flexibility as evidenced by longer OTW, shorter chest expansion, and Schober test than those with negative HLA-B27 (Table 1). Schober test [adjusted OR (aOR) 0.77, 95% CI 0.60–0.99] and log 10 transformation of OTW (logOTW) [aOR 5.16, 95% CI 1.34–19.84] remained statistically significant after adjustment for AS and log 10 transformation of disease duration (logdisease duration); however, chest expansion did not remain statistically significant [aOR 0.97, 95% CI 0.74–1.27]. The BASDAI, BASFI, and EQ-5D-5L indexes were all comparable between patients with positive and negative HLA-B27.

### Characteristics and factors associated with delayed diagnosis among axSpA patients

Median (interquartile range, IQR) diagnostic delay was 5.0 (1.7–11.1) years among axSpA patients. Positive HLA-B27, uveitis, history of good response to NSAIDs, and younger age at onset of the first musculoskeletal symptom were significantly associated with longer diagnostic delay, while psoriasis, first presentation with arthritis-enthesitis, IBP, and peripheral arthritis were significantly associated with shorter diagnosis delay in univariate linear regression analysis (Table 3). In multiple regression analysis, elevated CRP was significantly associated with shorter diagnostic delay, while uveitis and younger age at onset of first musculoskeletal symptom were significantly associated with longer diagnostic delay after adjustment for other variables. Conversely, HLA-B27, the first presentation with peripheral arthritis or enthesitis, IBP, peripheral arthritis, psoriasis, and good response to NSAIDs were not significantly associated with delayed diagnosis duration after adjustment for other variables.

**Table 3:** Factors associated with duration of delayed diagnosis in 177 patients with axial spondyloarthritis.

Characteristics	Univariate linear regression		Multiple linear regression	
	B	*p*-value	B	*p*-value
Female gender	− 0.059	0.359	NA	NA
First symptom age	− 0.013	< 0.001	− 0.010	0.002
HLA-B27	0.236	0.001	0.129	0.057
First axial symptom	0.096	0.157	NA	NA
First arthritis-enthesitis	− 0.173	0.006	− 0.102	0.110
Inflammatory back pain	− 0.171	0.043	− 0.077	0.373
Arthritis	− 0.162	0.024	− 0.092	0.171
Enthesitis	− 0.091	0.170	NA	NA
Dactylitis	− 0.075	0.300	NA	NA
Uveitis	0.338	< 0.001	0.269	< 0.001
Psoriasis	− 0.213	0.002	0.039	0.595
Good response to NSAIDs	0.129	0.038	0.100	0.122
Family history	− 0.039	0.569	NA	NA
Elevated C-reactive protein	− 0.118	0.063	− 0.144	0.012

log 10 transformation data.

*B* unstandardized coefficients, *Family history* presence in first-degree or second-degree relatives of any of the following: ankylosing spondylitis, psoriasis, uveitis, reactive arthritis, or inflammatory bowel disease, *First axial symptom* first presentation with inflammatory back pain and/or buttock pain, *First arthritis-enthesitis* first presentation with peripheral arthritis or enthesitis, *First symptom age* age at onset of the first musculoskeletal symptoms, *NA* not applicable, *NSAIDs* non-steroidal anti-inflammatory drug.

A *p*-value < 0.05 indicates statistical significance.

Linear regression analysis was used to identify significantly associated factors with the duration from the first musculoskeletal symptom to diagnosis; the dependent factor was the duration from the first musculoskeletal symptom to diagnosis.

Patients with longer diagnostic delay were more likely to have less spinal flexibility [logOTW (B = 0.018, *p* = 0.006), chest expansion (B =  − 0.054, *p* = 0.014), and a poorer Schober test result (B =  − 0.034, *p* = 0.062)] than those with shorter diagnostic delay. After adjustment for AS diagnosis, longer OTW was still significantly associated with longer diagnostic delay (logOTW: B = 0.216, *p* = 0.004), while chest expansion (B =  − 0.042, *p* = 0.058) and Schober test (B =  − 0.025, *p* = 0.171) had marginal association. Interestingly, EQ5D index (B = 0.301, *p* = 0.049) was positively associated with diagnostic delay in simple linear regression; however, it did not after adjusted for age (B = 0.23, *p* = 0.109). BASDAI (B =  − 0.012, *p* = 0.407) and BASFI (B =  − 0.009, *p* = 0.513) did not.

### Significant differences in clinical characteristics between AS and axial-PsA

Of a total of 177 axSpA [114 (64.4%) AS, 49 (27.7%) axial-PsA, and 14 (7.9%) uSpA], AS patients had the highest prevalence of positive HLA-B27 [94 (82.5%)] compared to uSpA [10 (71.4%)], and PsA [23 (46.9%)] (*p* < 0.001). AS patients were more likely to be younger at onset of the first musculoskeletal symptom [26.2 ± 8.4 *vs.* 33.5 ± 10.3 years, *p* < 0.001], younger at onset of IBP [27.5 ± 8.0 *vs.* 33.7 ± 9.1 years, *p* < 0.001], to have longer diagnostic delay [median (IQR), 6.5 (2.0–12.1) *vs.* 2.4 (1.0–5.2) years, *p* = 0.001], and lower mean number of SpA features excluding HLA-B27 [4.3 ± 1.5 *vs.* 5.3 ± 1.4, *p* < 0.001] than those with axial-PsA. In addition, AS patients had a higher frequency of axial involvement as the first symptom, IBP, uveitis, and good response to NSAIDs, as well as, less frequency of first presentation with arthritis, and dactylitis when compared to those with axial-PsA (Table 4). The proportion of female gender was comparable between AS and PsA patients. AS patients were more likely to have longer median (IQR) OTW [0 (0–6.6) *vs.* 0 (0–0) cm, *p* < 0.001], shorter mean chest expansion [3.1 ± 1.3 *vs.* 3.8 ± 1.5 cm, *p* = 0.004], and poorer Schober test result [3.7 ± 1.9 *vs.* 4.4 ± 1.3 cm, *p* = 0.021] than axial-PsA patients. However, none of these 3 factors were significantly different after adjustment for disease duration and HLA-B27 in multiple regression analysis (data not shown).

**Table 4:** Characteristics of ankylosing spondylitis and axial psoriatic arthritis subgroups, and factors associated with ankylosing spondylitis.

Characteristics	AS (n = 114)	PsA (n = 49)	Univariate regression OR (95% CI)	Multiple regression OR (95% CI)
Female gender, n (%)	42 (36.8%)	19 (38.8%)	0.82 (0.46–1.84)	NA
Age at first symptom (years), mean ± SD	26.2 ± 8.4	33.5 ± 10.3	0.92 (0.88–0.95)*	0.95 (0.90–0.99)**
First axial symptom, n (%)	90 (78.9%)	23 (46.9%)*	4.24 (2.07–8.70)*	3.93 (1.46–10.61)**
HLA-B27, n (%)	94 (82.5%)	23 (46.9%)*	5.31 (2.54–11.14)*	4.75 (1.80–12.53)**
Inflammatory back pain, n (%)	103 (90.4%)	39 (79.6%)	2.40 (0.95–6.10)	NA
Arthritis, n (%)	86 (75.4%)	44 (89.8%)**	0.35 (0.13–0.97)**	1.10 (0.32–3.77)
Enthesitis, n (%)	70 (61.4%)	36 (73.5%)	0.57 (0.28–1.20)	NA
Dactylitis, n (%)	18 (15.8%)	22 (44.9%)*	0.23 (0.11–0.49)*	0.15 (0.05–0.46)**
Uveitis, n (%)	36 (31.6%)	4 (8.2%)**	5.19 (1.74–15.54)**	6.79 (1.62–28.48)**
Good response to NSAIDs, n (%)	72 (63.2%)	14 (28.6%)*	4.29 (2.07–8.87)*	3.76 (1.47–9.61)**
Family history, n (%)	33 (28.9%)	17 (34.4%)	0.77 (0.38–1.57)	NA
Elevated C-reactive protein, n (%)	68/112 (60.7%)	30/49 (61.2%)	0.98 (0.49–1.95)	NA

Logistic regression analysis was used to identify factors significantly different between patients with ankylosing spondylitis and patients with psoriatic arthritis with axial involvement; the dependent factor was ankylosing spondylitis.

*AS* ankylosing spondylitis, *95% CI* 95% confidence interval, *Family history* presence in first-degree or second-degree relatives of any of the following: ankylosing spondylitis, psoriasis, uveitis, reactive arthritis, or inflammatory bowel disease, *First axial symptom* first presentation with inflammatory back pain and/or buttock pain, *NA* not applicable, *NSAIDs* non-steroidal anti-inflammatory drugs, *OR* odds ratio, *PsA* psoriatic arthritis with axial involvement.

*p-value < 0.001; **p-value < 0.05–0.001.

In univariate regression analysis, first presentation with axial symptoms, positive HLA-B27, uveitis, and good response to NSAIDs were significantly positively associated with AS, while age at onset of the first musculoskeletal symptom, arthritis, and dactylitis were significantly negatively associated with AS. Multiple regression analysis revealed positive HLA-B27, uveitis, good response to NSAIDs, first presentation with axial symptom to be positively associated with AS, and age at onset of the first musculoskeletal symptom and dactylitis was negatively associated with AS after adjustment for other variables.

## Discussion

This study found a prevalence of positive HLA-B27 of 71.8% for axSpA, 82.5% for AS, and 46.9% for axial-PsA patients, while the prevalence of HLA-B27 in Thai general population was estimated to be 4–7%^11,19,25,26^. The prevalence in our study was comparable to previously reported rates from countries that have comparable HLA-B27 frequency in general population (2–9%)^6^, such as 62% among French combined nr-axSpA and r-axSpA patients^8^, 72.3% among Chinese nr-axSpA patients^27^, and 82% among German AS patients^24^. In contrast, the prevalence among axSpA in the Middle East and among Arab AS patients varies widely from 26% in Lebanon to 91% in Turkey^5,28^, and the HLA-B27 frequency in general population in these countries ranges from 3 to 14%^6^. Moreover, there was a higher frequency of HLA-B27 among r-axSpA than among nr-axSpA^24^, and AS had a higher prevalence of HLA-B27 than any other SpA diseases^6^, which is consistent with the present study. In addition, Deesomchok et al. reported a prevalence of HLA-B27 among 56 Thai AS of 92%^29^, which is higher than the current study. This may be due to the fact that the Deesomchok et al. study included a higher proportion of patients with younger age at onset than the current study. Positive HLA-B27 status was significantly associated with younger age at onset of IBP in the current study, and in other previous reports from Germany^24^ and Finland^30^. Regarding axial-PsA, the prevalence of positive HLA-B27 in the present study was comparable to previous study in axial-PsA in the United Kingdom (40%)^31^. The prevalence of positive HLA-B27 in PsA in the present study was lower than in the Deesomchok et al. study (60%), which included only 10 PsA patients^11^.

There is variation in the characteristics that have been reported to associate with HLA-B27 status in axSpA patients^5,7,8^. In the current study, patients with HLA-B27 were positively associated with uveitis and younger age at onset of IBP, which is similar to previous studies in axSpA^8^ and AS^7^ patients. Moreover, positive HLA-B27 status was negatively associated with female gender and psoriasis, which is similar to the findings of Chung et al.^8^. Conversely, arthritis, enthesitis, and relevant family history had no association with HLA-B27, which differed from the Chung et al. study^8^. These characteristics were not found to significantly associate with HLA-B27 in studies conducted in Turkey^32^ and Lebanon^28^. This may be due to different duration of disease, ethnicity, and/or genetics, including HLA-B27 subtype. Chung et al. reported Caucasian race to be associated with older age at onset of IBP^8^. The present study found AS status and longer disease duration to be associated with decreased spinal flexibility. This may be due to structural damage, which was previously reported^33^. Moreover, we found axSpA with positive HLA-B27 to be more likely to have less spinal flexibility (longer OTW and shorter SchÖber test) than those with negative HLA-B27 after adjustment for AS status and disease duration. HLA-B27 may be a factor that is significantly associated with severity among axSpA; however, this hypothesis needs to be confirmed in future study.

AS and axial-PsA are sometimes difficult to differentiate. Jadon et al. reported disease activity, metrology, and disability to be comparable between axial-PsA and AS^31^; however, AS had a higher frequency of positive HLA-B27, and greater evidence of structural damage from sacroiliitis and spondylitis^10,31^. Axial-PsA patients were older at onset of back pain, and had a higher frequency of asymptomatic status and female gender than AS patients^10,34^. Aydin et al. reported that female axial-PsA patients were less likely to fulfill IBP criteria^35^. This study recruited only symptomatic axSpA, and demonstrated HLA-B27, first presentation with axial symptom, age at onset of the first musculoskeletal symptom, good response to NSAIDs, uveitis, and dactylitis to be associated with AS after adjustment for other variables excluding psoriasis. Male gender did not differ between AS and axial-PsA in this study, which is different from the previous reports^10,31^. This may be due to this study included only symptomatic patients with chronic back pain which was different from previous study^31^.

Diagnostic delay was reported to be associated with poor outcome^2,3^. The current study found decreased cervical flexibility to be associated with longer diagnostic delay after adjustment for AS status. Interestingly, the EQ5D Index was positively associated with longer delay in diagnosis. Patients with a lower EQ5D Index may have greater disease activity, so they may seek health care and be diagnosed earlier than those with less active disease. Our study also found elevated CRP to be associated with shorter diagnostic delay, and elevated CRP associated with lower EQ5D Index (B =  − 0.066, *p* = 0.04). Delayed diagnosis is one of the main problems in AS and maybe axSpA because IBP demonstrates no visual sign of inflammation that can be detected by physical examination like peripheral arthritis. IBP is diagnosed by taking history of IBP character. The proportion of Thai physicians that knew all of the features of IBP was reported to be only 25%^36^. Moreover, not all IBP patients are axSpA^37^. In our study, younger age at onset of the first symptom was associated with longer delay in diagnosis, while positive HLA-B27 status was not after adjustment for other variables. This is in contrast to the findings of Feldtkeller et al. who conducted a study in AS patients^38^. HLA-B27 testing is not commonly performed in routine clinical practice among patients suspected of having AS in Thailand because it is not required for diagnosis, and it is a special and not widely available test. We performed HLA-B27 testing when patients had clinically suspected axSpA (as addressed in the “Materials and methods” section) regardless of the duration of disease in this study; therefore, HLA-B27 in this study may not reflect association with delayed diagnosis. However, HLA-B27 may be an important biomarker to classify axSpA in the early course of the disease because it was shown to have strong association with AS and axSpA in both low and high prevalence HLA-B27 countries^5,7^ among suspected axSpA patients, including in Thailand as shown in the current study.

Anterior uveitis was also found to be associated with longer diagnostic delay. In contrast, Yacoub et al. conducted a study in Morocco and did not find association between diagnostic delay and age at onset or extra-articular manifestation^39^. Most studies found female patients to be more likely to have longer disease duration than males despite the presence of other conflicting data^40^. Our study did not find association between gender and delayed diagnosis. To early diagnose axSpA, knowledge of axSpA characteristics should be introduced to ophthalmologists and other physicians, and a referral strategy for early diagnosis of axSpA should be developed as previous reports^41,42^. Moreover, HLA-B27 testing should be performed in patients suspected of having axSpA without evidence of radiographic sacroiliitis^22^, and in young patients with IBP and without suspected other diseases because it may decrease diagnostic delay. Moreover, it is less expensive and easier to interpret than performing MRI of the pelvis in Thailand. The utility of and indications for HLA-B27 investigation for axSpA diagnosis in Thailand requires further study.

This study has some limitations. First, this was a cross-sectional study, which means that the disease could change during the course of disease. Longitudinal study is, therefore, needed. Second, all included patients came from a single tertiary care hospital that is routinely referred complex cases. Moreover, a few axial SpA patients did not have both HLA-B27 and pelvic radiography, so they were excluded from our study. It is unclear whether our findings are generalizable to all Thai axSpA patients. Finally, none of our study patients underwent sacroiliac joint MRI; therefore, there were few nr-axSpA patients. Performing MRI in this setting in Thailand is not currently practical. Improved knowledge about this disease and improved MRI availability are both needed. As such, this study reflects current clinical practice in Thailand. The strength of this study is that clinical data were collected by reviewing medical records and interviewing patients face to face, and musculoskeletal examination was fully performed in every patient.

## Conclusion

The prevalence of positive HLA-B27 was 71.8% among Thai axSpA patients. Male gender, age of onset of the first musculoskeletal symptom of ≤ 28 years, uveitis, and psoriasis were associated with HLA-B27 in axSpA patients. The median delay in diagnosis of axSpA was approximately 5 years. Younger age at onset, uveitis, and normal CRP were associated with longer diagnostic delay. Young patients with musculoskeletal symptoms and patients with uveitis should be considered for axSpA screening. Criteria for referral and HLA-B27 testing may decrease delay in diagnosis; however, further studies are needed to prove the efficacy cost effectiveness of these proposed improvements in Thailand.

## References

[1] Zhao SS (2021). Diagnostic delay in axial spondyloarthritis: A systematic review and meta-analysis. Rheumatology (Oxford).

[2] Bakland G, Gran JT, Nossent JC (2011). Increased mortality in ankylosing spondylitis is related to disease activity. Ann. Rheum Dis..

[3] Haroon M, Gallagher P, FitzGerald O (2015). Diagnostic delay of more than 6 months contributes to poor radiographic and functional outcome in psoriatic arthritis. Ann. Rheum. Dis..

[4] Bowness P (2015). Hla-B27. Annu. Rev. Immunol..

[5] Ziade NR (2017). HLA B27 antigen in Middle Eastern and Arab countries: Systematic review of the strength of association with axial spondyloarthritis and methodological gaps. BMC Musculoskelet. Disord..

[6] Khan MA (2008). HLA-B27 and its pathogenic role. J. Clin. Rheumatol..

[7] Lin H, Gong YZ (2017). Association of HLA-B27 with ankylosing spondylitis and clinical features of the HLA-B27-associated ankylosing spondylitis: A meta-analysis. Rheumatol. Int..

[8] Chung HY, Machado P, van der Heijde D, D'Agostino MA, Dougados M (2011). HLA-B27 positive patients differ from HLA-B27 negative patients in clinical presentation and imaging: Results from the DESIR cohort of patients with recent onset axial spondyloarthritis. Ann. Rheum. Dis..

[9] Helliwell PS (2020). Axial disease in psoriatic arthritis. Rheumatology (Oxford).

[10] Feld J (2020). Is axial psoriatic arthritis distinct from ankylosing spondylitis with and without concomitant psoriasis?. Rheumatology (Oxford).

[11] Deesomchok U, Tumrasvin T, Bejraputra O (1983). HLA-B27 in Thai patients with arthritis. J. Med. Assoc. Thai..

[12] Rudwaleit M (2009). The development of Assessment of SpondyloArthritis international Society classification criteria for axial spondyloarthritis (part II): Validation and final selection. Ann. Rheum. Dis..

[13] Kittiyanpanya C, Chaiamnuay S, Asavatanabodee P, Narongroeknawin P (2014). Reliability and validity of the Thai version of bath ankylosing spondylitis indices. J. Med. Assoc. Thai..

[14] Oemar M, Janssen B (2013). EQ-5D-5L User Guide: Basic Information on How to Use the EQ-5D-5L Instrument.

[15] Pattanaphesaj, J. Health-related quality of life measure (EQ-5D-5L): Measurement property testing and its preference-based score in Thai population [Doctoral dissertation]. Doctoral thesis, Mahidol (2014).

[16] Maksymowych WP (2009). Development and validation of the Spondyloarthritis Research Consortium of Canada (SPARCC) Enthesitis Index. Ann. Rheum. Dis..

[17] Heuft-Dorenbosch L (2003). Assessment of enthesitis in ankylosing spondylitis. Ann. Rheum. Dis..

[18] Sieper J (2009). The Assessment of SpondyloArthritis international Society (ASAS) handbook: A guide to assess spondyloarthritis. Ann. Rheum. Dis..

[19] Chandanayingyong D (1997). HLA-A, -B, -DRB1, -DQA1, and -DQB1 polymorphism in Thais. Hum. Immunol..

[20] Hopkins, A. K. In *American Society for Histocompatibility and Immunogenetics,* Vol. 1 (eds B. Amy. Hahn, B. A. *et al*.) 1–7 (2000).

[21] van der Heijde DM (2009). New criteria for inflammatory back pain in patients with chronic back pain—A real patient exercise of the Assessment in SpondyloArthritis international Society (ASAS). Ann. Rheum. Dis..

[22] van der Linden S, Valkenburg HA, Cats A (1984). Evaluation of diagnostic criteria for ankylosing spondylitis. A proposal for modification of the New York criteria. Arthritis Rheum..

[23] Taylor W (2006). Classification criteria for psoriatic arthritis: Development of new criteria from a large international study. Arthritis Rheum..

[24] Rudwaleit M (2009). The early disease stage in axial spondylarthritis: Results from the German Spondyloarthritis Inception Cohort. Arthritis Rheum..

[25] Romphruk AV (2010). HLA class I and II alleles and haplotypes in ethnic Northeast Thais. Tissue Antigens.

[26] Duangchanchot M (2009). HLA-B*27 subtypes in Northern and Northeastern Thais, Karens, and Bamars determined by a high-resolution PCR-SSP technique. Tissue Antigens.

[27] Lin Z (2014). Evaluation of Assessment of Spondyloarthritis International Society classification criteria for axial spondyloarthritis in Chinese patients with chronic back pain: Results of a 2-year follow-up study. Int. J. Rheum. Dis..

[28] Ziade N (2019). HLA-B27 prevalence in axial spondyloarthritis patients and in blood donors in a Lebanese population: Results from a nationwide study. Int. J. Rheum. Dis..

[29] Deesomchok U, Tumrasvin T (1985). Clinical study of Thai patients with ankylosing spondylitis. Clin. Rheumatol..

[30] Jaakkola E (2006). Finnish HLA studies confirm the increased risk conferred by HLA-B27 homozygosity in ankylosing spondylitis. Ann. Rheum. Dis..

[31] Jadon DR (2017). Axial disease in psoriatic arthritis study: Defining the clinical and radiographic phenotype of psoriatic spondyloarthritis. Ann. Rheum. Dis..

[32] Firat SN (2017). Low frequency of HLA-B27 in ankylosing spondylitis and its relationship with clinical findings in patients from Turkey. Eur. J. Rheumatol..

[33] Ramiro S (2014). Higher disease activity leads to more structural damage in the spine in ankylosing spondylitis: 12-year longitudinal data from the OASIS cohort. Ann. Rheum. Dis..

[34] Feld J, Chandran V, Haroon N, Inman R, Gladman D (2018). Axial disease in psoriatic arthritis and ankylosing spondylitis: A critical comparison. Nat. Rev. Rheumatol..

[35] Aydin SZ, Kilic L, Kucuksahin O, Ureyen SB, Kalyoncu U (2017). Performances of inflammatory back pain criteria in axial psoriatic arthritis. Rheumatology (Oxford).

[36] Tangrungruengkit M, Srinonprasert V, Chiowchanwisawakit P (2016). Survey of Thai physicians regarding recognition and management of inflammatory back pain and spondyloarthritis. J. Med. Assoc. Thai..

[37] Bakland G, Alsing R, Singh K, Nossent JC (2013). Assessment of SpondyloArthritis International Society criteria for axial spondyloarthritis in chronic back pain patients with a high prevalence of HLA-B27. Arthritis Care Res. (Hoboken).

[38] Feldtkeller E, Khan MA, van der Heijde D, van der Linden S, Braun J (2003). Age at disease onset and diagnosis delay in HLA-B27 negative vs. positive patients with ankylosing spondylitis. Rheumatol. Int..

[39] Ibn Yacoub Y, Amine B, Laatiris A, Bensabbah R, Hajjaj-Hassouni N (2012). Relationship between diagnosis delay and disease features in Moroccan patients with ankylosing spondylitis. Rheumatol. Int..

[40] Rusman T, van Vollenhoven RF, van der Horst-Bruinsma IE (2018). Gender differences in axial spondyloarthritis: Women are not so lucky. Curr. Rheumatol. Rep..

[41] Garcia Salinas R (2021). “Reuma-Check”: Performance of a comprehensive fast-track program for the diagnosis of axial spondyloarthritis in South America. J. Clin. Rheumatol..

[42] Adshead R, Tahir H, Donnelly S (2015). A UK best practice model for diagnosis and treatment of axial spondyloarthritis. EMJ Rheumatol..

